# Differences on the Natural Course of Chronic Kidney Disease Progression, Induced by 5/6 Renal Ablation Model in Three Different Rat Stains: Wistar, Lewis, and Fischer 344

**DOI:** 10.3390/life16030420

**Published:** 2026-03-04

**Authors:** Samuel de Jesus, Paloma Souza Noda, Ana Laura Rubio Francini, Flavio Teles Filho, Mariana Matera Veras, Ane Claudia Fernandes Nunes, Irene de Lourdes Noronha, Camilla Fanelli

**Affiliations:** 1 Laboratory of Cellular, Genetic, and Molecular Nephrology, Renal Division, Faculty of Medicine, University of São Paulo, São Paulo 01246-903, SP, Brazil; 2 Renal Division, Faculty of Medicine, Federal University of Alagoas, Maceio 57200-000, AL, Brazil; 3 Laboratory of Environmental and Experimental Pathology, Department of Pathology, School of Medicine, University of São Paulo, São Paulo 01246-903, SP, Brazil; 4 Division of Nephrology, Hypertension and Kidney Transplantation, School of Medicine, University of California, Irvine, CA 92617, USA; 5 Nova Friburgo Health Institute, Fluminense Federal University, Nova Friburgo 28625-650, RJ, Brazil

**Keywords:** chronic kidney disease, experimental model, nephron number, 5/6 renal ablation

## Abstract

Almost 10% of the global population suffers from chronic kidney disease (CKD). The inexistence of a therapeutic to restore renal function motivates the scientific community to search for new treatments. The 5/6 nephrectomy (Nx) rat model is widely used to mimic human CKD, but the impact of strain-specific responses on disease progression remains unclear. Here, we aimed to compare CKD development in Wistar, Lewis, and Fischer rats submitted to the Nx model. In summary, even submitted to the same surgical procedure, the three studied rat strains presented distinct patterns of CKD progression: Wistar rats exhibited faster and sustained renal function loss, with exuberant hypertension, proteinuria, and renal inflammation, being considered as excellent models to study rapidly progressive human nephropathy. Lewis animals, in turn, presented mild low-progressive CKD, which make this rat strain especially useful to simulate intermediate degrees of human CKD and to develop long-term tests. Finally, Fischer rats submitted to Nx did not even develop hypertension, proteinuria, or glomerular damage within 30 days. Moreover, compared to Wistar rats, both Lewis and Fischer animals have a relatively higher basal number of nephrons and a lower number of whole blood leukocytes, which may have contributed to the renoprotection exhibited by these rat strains.

## 1. Introduction

Chronic kidney disease (CKD) is a severe and highly debilitating condition, characterized by the progressive reduction of renal function, persisting for a minimum of three months, by the development of albuminuria, and by the presence of kidney structural damage, leading to the necessity of life-sustaining renal replacement therapy (RRT) [1,2]. According to the most recent epidemiological surveys, the total number of adult patients with CKD in 2023 was approximately 788 million worldwide, representing about 9.5% of the global population. Moreover, CKD was the ninth leading cause of death around the world, being considered a major global health issue [3].

Among many essential functions, the kidneys are responsible for the hydroelectrolytic and the acid-base balance of the organism, thus exerting a core function to the maintenance of homeostasis. Human kidneys are composed of approximately two million of interdependent filtration units called nephrons. Together, the whole set of nephrons filters blood plasma through their glomeruli and processes the filtered metabolites in the renal tubules, thus promoting the excretion of toxic elements and the controlled reabsorption of ions, electrolytes, and water. Renal function is usually evaluated through the glomerular filtration rate (GFR), which results from the multiplication of each single nephron GFR by the total number of nephrons in both kidneys. Therefore, the loss of nephrons, which can be caused by a myriad of different factors, such as persistent hypertension, imbalanced diabetes, genetic conditions, and autoimmune diseases, among others, directly impacts the whole organ performance.

The maladaptive repair of the kidney after an initial renal insult, which promotes the first loss of filtrating units, can be considered the core factor for the development of CKD. Nephron number reduction leads to the insidious overload of remaining units, which develop compensatory hyperfiltration and hypertrophy in order to deal with the entire cardiac output. These changes, along with the hyperactivity of both systemic and intra-renal renin–angiotensin–aldosterone system (RAAS), lead to the development of local inflammation, which progresses to tissue fibrosis and to the loss of even more nephrons, resulting in the failure of renal function [4,5,6].

In the last ten years, some great advances in the treatment of CKD have been seen. Associated with lifestyle change recommendations, the employment of pharmacological therapies with angiotensin-converting enzyme inhibitors (ACEi), angiotensin receptor blockers (ARB), and, most recently, SGLT2 inhibitors, have shown great efficacy in slowing down CKD progression. However, such strategies are still not able to completely prevent or reverse renal function loss. Most CKD patients may still require supportive RRT later in life [1].

The lack of a specific treatment to stunt CKD and restore renal function motivates physicians and the scientific community to continue studying the mechanisms involved with CKD progression, in order to develop new experimental and preclinical strategies for CKD detaining. This highlights the importance of animal-based experimental studies, which serve as the basis for verifying the safety and efficacy of new treatments [7].

The rat (*Rattus norvegicus*) represents one of the most commonly employed species in the development of preclinical studies in search of innovative CKD treatments, once its kidney physiology closely resembles human features. Rats for research purposes can be classified as outbred; obtained through a breeding system with no mating of close relatives, or inbred; produced from at least 20 consecutive generations of brother–sister mating. The main difference between these two breeding systems is the genetic variance of the produced population. An outbred rat stock is characterized by remarkable diversity among individuals, due to the highly variable genetic background, while an inbreed rat strain is composed of genetically uniform animals. Wistar and Sprague–Dawley strains are among the most popular outbreed rats used experimentally for renal pathophysiology research worldwide, while Lewis and Fischer inbreed stocks are used in experimental renal transplantation studies [8,9,10,11,12].

Human CKD can be mimicked in rats through different experimental methodologies, among which, the sub-total nephrectomy model, achieved through the 5/6 renal ablation, stands out. There are at least two different ways to perform this model, but concisely, it consists of the surgical removal of the right kidney, combined with the reduction of left kidney mass by 2/3, thus promoting a sudden reduction in the functioning nephron number, leading to the development of rapidly progressive CKD. The 5/6 renal ablation model, the so-called remnant model, or simply “Nx”, is widely known to closely resemble the clinical settings observed in human nephropathy, as the development of systemic hypertension, albuminuria, uremia, and renal structural changes, such as glomerulosclerosis, renal interstitial fibrosis, and inflammation [13,14,15,16,17].

Although, compared to other rodents, rats are the species of choice when applying the Nx CKD model, until the present moment, there is no consensus about the most appropriate rat strain for this purpose. The current literature indicates that approximately 60% of studies employing the 5/6 renal ablation model of CKD use Sprague–Dawley rats, while 39% use Wistar rats, and less than 1% use other rat strains. Although Lewis and Fischer strains are widely used in renal allograft rejection studies, their application in the 5/6 ablation model remains under-explored. As isogenic strains, Lewis and Fischer animals require constant breeding to maintain specific genetic traits, often resulting in surplus animals. Repurposing these animals for CKD research aligns with ethical considerations regarding animal welfare and resource optimization. However, the literature lacks a systematic, head-to-head comparison of CKD development and evolution among these inbreed rat strains and a traditionally used outbreed strain, within a standardized 5/6 nephrectomy protocol [18,19,20,21].

Given that the biological responses of Lewis and Fischer rats to this model are not yet fully characterized, we hypothesize that, despite similar body weight, Wistar, Lewis, and Fischer rats may exhibit distinct trajectories of CKD progression when submitted to the 5/6 renal ablation model, which can be dependent on genetic and phenotypic variations and on inherent differences in nephron endowment between the studied rat strains [22,23,24,25].

Of note, there is a significant knowledge gap with regard to which rat strain is the most appropriate for each kind of preclinical study in the nephrology field: For longer studies, in which multiple periodical applications of a specific therapy need to be evaluated, for instance, using an experimental model of slower-progressing CKD may be more interesting, because it allows the comparison between treated and untreated animals, with a low risk of losing the whole untreated group due to the disease worsening and the high mortality rates associated with rapidly progressive models. On the other hand, for time-limited studies, in which a single therapy application will be tested or a smaller time course of follow-up is needed, rapidly progressive rat models of CKD may be preferred [13,16].

On this basis, the aim of this study was to compare the severity and the rhythm of nephropathy progression in three different rat strains: Wistar, Lewis, and Fischer, submitted to the 5/6 renal ablation model of CKD. Elucidating these strain-specific responses is essential to refine experimental models and ensure the reproducibility of findings.

## 2. Material and Methods

### 2.1. Animals and CKD Model

Wistar, Lewis, and Fischer rats of the species *Rattus norvegicus* employed in this study were purchased from established colonies of the Central Animal Facility of the University of São Paulo. In order to define the appropriate number of animals to be used in each experimental group, to ensure the statistical relevance of the obtained results, we applied the sample size (SS) calculation equation for comparison between two groups, with a quantitative outcome, as follows: SS = 2 SD^2^ (Zα/2 + Zβ) 2/d^2^ [26,27,28]. This calculation was based on the expected variability between control and model animals, for the main outcomes of our study, including functional (systolic blood pressure, proteinuria, albuminuria, serum creatinine, and urea) and histopathological parameters (glomerular lesion score and interstitial fibrosis), obtained from previous studies employing Wistar rats submitted to the 5/6 renal ablation model (Nx). From these previous papers, we extracted both the standard deviation (SD) and the effect size (d), obtained from the comparison between control and Nx Wistar rats [29,30]. We considered an acceptable value of 5% for type 1 error (α) and 20% for type 2 error (β), with a statistical power of 80% (generally adequate for biological studies using animal models); thus, “Zα” and “Zβ” were constant numbers obtained from the Z table when α = 5% and β = 20%, respectively. The obtained SS was subjected to further correction, considering that the expected mortality rate of 20% for rats underwent 5/6 renal ablation, in the first 30 days of follow-up, as follows: Corrected SS = SS/(0.8), thus resulting in a minimum of 16 animals per group, by the end of the study period [26,27,28,29,30].

Therefore, 107 adult male rats—36 Wistar (outbred animals), 36 Lewis, and 35 Fischer 344 (inbred)—were housed in appropriate cages, with maximum allocation of 3 rats per cage, and kept in the local animal facility of the Renal Division of the University of São Paulo School of Medicine, under controlled conditions: room temperature of 23 ± 1 °C; relative air humidity of 60 ± 5%; and light/dark 12 h/12 h cycle. Rats had free access to drinking water and conventional rodent food (Nuvital Nutrientes, Colombo, PR, Brazil) throughout the study period, and were included in the experimental groups when the mean body weight of 220~280 g was achieved. All experiments described in this study were analyzed and approved by the ethics committee for the use of experimental animals of the Faculty of Medicine of the University of São Paulo (CEUA FMUSP protocols 1018/2018 and 2003–2023).

CKD was induced in 19 Wistar, 18 Lewis, and 17 Fischer animals, through the 5/6 renal ablation model (Nx), in a single step. For this purpose, rats were anesthetized by inhalation with 5% isoflurane (Instituto BioChimico, Rio de Janeiro, Rj, Brazil) for anesthesia induction, and 3% for maintenance of anesthesia. After achieving complete unconsciousness, the animals were placed on heated surgical tables, where a physiological temperature of 37 °C was maintained throughout the whole procedure. After anesthetizing the rats, sensitivity tests were performed with rat tooth tweezers forceps, and the animals were submitted to ventral trichotomy and median laparotomy under aseptic conditions. The left kidney was carefully exposed and hydrated with sterile saline solution. Under a final 16× magnification, using an appropriate microsurgery microscope, we performed the ligation of 2 of the 3 branches of the renal artery of the left kidney, with silk 6.0 needled suture, thus promoting the infarction of 2/3 of the organ, immediately detectable by the purplish color assumed by the whole posterior side of the kidney and by the lower half of the anterior side of it. The left kidney was replaced into the abdominal cavity, and the right kidney was exposed. Using a curved Kelly artery forceps, we isolated the right kidney artery and vein and permanently ligated the vascular branch using cotton 3.0 sterile suture, to avoid intraoperative bleeding. The right kidney was then removed and discarded, resulting in the total reduction of 5/6 of the rat renal mass. The remaining 17, 18, and 18 animals of each rat strain, respectively, were used as controls. These animals were also anesthetized and subjected to a median laparotomy; however, with no ablation or removal of renal mass (Wistar Sham, Lewis Sham, and Fischer Sham). Immediately after surgery, all rats received intramuscular (IM) injections of prophylactic antibiotic (5 mg/kg Baytril, Bayer, São Paulo, SP, Brazil) and subcutaneous (SC) injections of analgesic (10 mg/kg Tramadol) and were kept in heated cages until they fully recovered from anesthesia. SC analgesic administration was repeated every 8 h for 2 more days.

### 2.2. Experimental Protocol and Sample Collection

Animals had their body weight (BW, g) monitored weekly, and their systolic blood pressure (SBP, mmHg) verified in 3 different moments of the protocol: At the beginning of the study, before CKD induction (0 days), and at 15 and 30 days after CKD induction. SBP was assessed through the caudal blood pressure method, using the automated optoelectronic device BP-2000 (Visitech Systems, Apex, NC, USA). Before each point of SBP acquisition, at 0, 15, and 30 days, all rats were submitted to an adaptation protocol of 3 days of mock repeated measurements, to ensure animals ambience and calm during the definitive data collection. At the same time-points (before CKD induction, and 15 and 30 days after CKD induction), rats were housed for 24 h in individual metabolic cages for urine samples collection. The urinary protein excretion rate (UPE, mg/24 h) was analyzed through a colorimetric method, employing a commercially available kit (SENSIPROT Kit #36, Labtest Brasil, Lagoa Santa. MG, Brazil), while the urinary albumin excretion rate (UAE, mg/24 h) was assessed by radial immunodiffusion using a specific anti-rat albumin antibody (anti-rat albumin MP-BIOMEDICALS, Irvine, CA, USA #55711).

Rats were followed for a total period of 30 days after CKD induction, when they were euthanized for blood and renal tissue samples collection. For this purpose, the animals were once more anesthetized with isoflurane, underwent midline laparotomy, and an abdominal aortic puncture for total blood collection, resulting in euthanasia by exsanguination. Blood samples were collected into two distinct tubes, one of which contained EDTA as an anticoagulant for hematological assessment. Total leukocyte counts were determined using an automated hematology analyzer (Horiba ABX, Horiba Medical, Montpellier, France). To ensure maximum precision, blood smears were subsequently prepared and stained with rapid Romanowsky stain for manual morphological analysis under light microscopy. This combined approach allowed for the accurate quantification of neutrophils, lymphocytes, monocytes, eosinophils, and basophils, expressed as absolute values (cells/mm^3^) and relative percentages. The other collection tube, containing clot activator, was centrifuged for 15 min at 2000 RPM at room temperature to obtain serum samples, from which we measured the urea (SUr, mg/dL) and creatinine concentrations (SCr mg/dL) through a colorimetric technique, using commercial kits (UREA CE Labtest Kit #27/CREATININE Labtest Kit #35). In order to calculate the creatinine clearance of animals, we employed the same colorimetric kit used for serum dosages and measured the creatinine concentration in the urine samples of animals after 30 days of CKD induction. Collected values were applied to the following formula: [(UCr × Urinary Volume)/SCr]/1440, thus obtaining the rate in mg/min. These values were than divided by the body surface area of each respective animal, obtained by multiplying the individual BW by the Meeh constant (k) of the rat (9.83), and final creatinine clearance (CrCl) was presented as mg/min/m^2^ [31].

After confirming the euthanasia of experimental animals, the left kidneys were collected, weighed, and transversely sectioned into two equal portions. The first half was subjected to Duboscq-Brazil prefixation for 30 min, followed by permanent fixation in buffered formaldehyde (pH 7.4) for 48 h. The right kidneys of Sham animals were collected, weighed, sectioned in sagittal direction, and formalin-fixed, for further use in the total counting of nephron number in each rat strain.

### 2.3. Histological and Immunohistochemical Analyses

Formaldehyde-fixed left kidney samples were processed for dehydration, clearing, and paraffin impregnation in the automatic tissue processor Leica TP1020 (Leica Biosystems, Wetzlar, Germany). Samples were then included in paraffin blocks in the inclusion station Leica Histocore Arcadia H; these blocks were used to produce slides with 5 µm histological sections, employed for histological and immunohistochemical analysis.

The periodic acid-Schiff (PAS) histological staining was used to evaluate the percentage of glomerulosclerosis in the renal slides of experimental animals, evidenced in magenta. The histological slides were deparaffinized, rehydrated, and immersed in a 1% periodic acid solution for 30 min. After rinsing in tap water, the slides were incubated in Schiff’s reagent for 60 min, protected from light in an amber container. Subsequently, the sections were counterstained with Carazzi’s hematoxylin, dehydrated in increasing concentrations of ethanol, and cleared in xylene. Finally, the slides were coverslipped using a synthetic mounting medium for subsequent microscopic analysis. At least 50 different glomeruli for each experimental animal were microphotographed, under a final 400× magnification. The pictures were coded and analyzed by a blind pathologist. Based on the assessment of each glomerulus, as sclerotic or non-sclerotic, we calculated the percentage of affected glomeruli.

To analyze the renal interstitial fibrosis, we used Masson’s Trichrome staining, which stains collagen deposits in blue. After deparaffinization and rehydration, the sections were immersed in Weigert’s iron hematoxylin for 10 min for nuclear staining. The slides were rinsed in running water and incubated in Biebrich scarlet solution for 10 min. Following a subsequent water rinse, the sections were treated with phosphotungstic–phosphomolybdic acid solution as a differentiator for 5 min. Finally, the slides were immersed in aniline blue solution to stain the collagen fibers, rinsed, dehydrated, and coverslipped with synthetic mounting medium. To quantify the percentage of affected area, all slides were coded, to ensure blinding, and observed under final 200× magnification. We analyzed 25 consecutive microscopic fields of each experimental animal, using a grid of equidistant points. Additionally, in order to complement the analysis of interstitial fibrosis, immunohistochemistry for α-smooth muscle actin (α-SMA) was performed in the renal samples of experimental animals, using a specific monoclonal anti-α-SMA antibody (Sigma-Aldrich (St. Louis, MO, USA), #A2547 diluted 1:1000). We assessed the percentage of tubulointerstitial area occupied by this protein as an indirect parameter for the presence of myofibroblasts, through the same point-counting methodology used to assess interstitial fibrosis by Masson’s staining.

Renal inflammation was assessed by both the interstitial macrophage infiltration and the increase in the interstitial cell proliferating rate, employing immunohistochemistry, with the specific antibodies anti-CD68 (Serotec (Bio-Rad Laboratories, Hercules, CA, USA), #MCA341R, diluted 1:200) and anti-PCNA (Proliferating Cell Nuclear Antigen, Dako (Dako, Glostrup, Denmark), #M0879 diluted 1:1000), respectively. After the codification of the slides, for each marker, positive cells were counted in 25 non-overlapping microscopic fields of each experimental animal, at a final 400× magnification and the results were expressed as cells/mm^2^ of renal tissue.

### 2.4. Total Nephron Number and Glomerular Measurements

**Figure 1:**
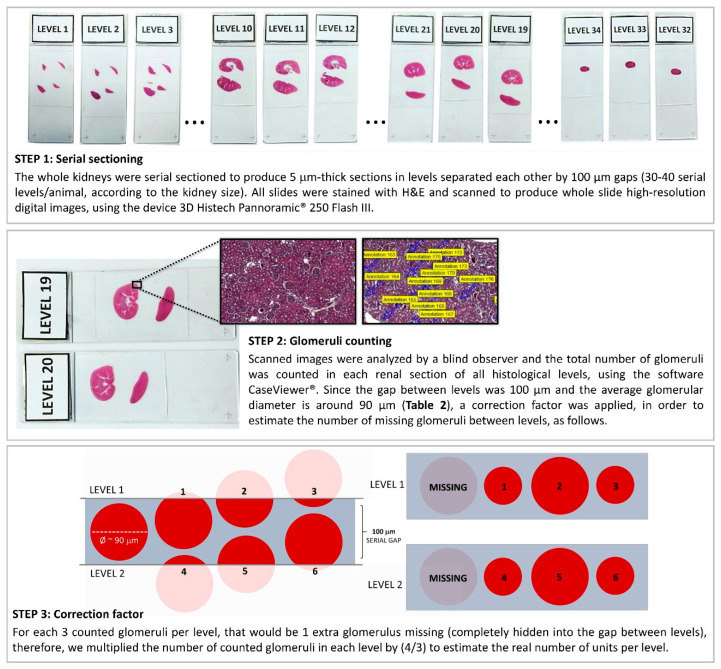
Illustrative scheme of the method employed to estimate the total nephron number in the studied rat strains. After counting the number of glomeruli in all renal sections of the right kidney of each animal, and multiplying this number by the correction factor (4/3), as illustrated, the obtained value was further multiplied by 2, in order to estimate the total Nephron number (Nn) per rat, in both kidneys.

The right kidneys of 5 Sham animals from the different rat strains were processed, included in histological paraffin blocks with the sectioned portion facing down, and submitted to serial sectioning to produce slides with 5 µm sections of renal tissue, every 100 µm of distance, until the tissue was completely consumed. Approximately 30–40 “levels” of histological sections were obtained from each experimental animal. The obtained renal slides were stained with hematoxylin and eosin (H&E). Briefly, the sections were deparaffinized, rehydrated, and immersed in Mayer’s hematoxylin for 4 min. After rinsing in Tris-buffered saline (TBS) to promote nuclear bluing, the slides were stained with 0.5% yellowish eosin for 2 min. Following a final wash in water, the sections were dehydrated, coverslipped, and scanned with the 3D Histech Pannoramic^®^ 250 Flash III equipment (3DHISTECH Ltd., Budapest, Hungary). All the obtained images were coded before evaluation, to ensure a blind analysis. The total number of glomeruli was counted, to achieve the total nephron number (Nn) of each rat strain, as illustrated in Figure 1. Moreover, for more precise comparisons between the different strains, we divided the Nn by the adult rat body weight (ARW), in grams, which is the mean body weight of each rat strain at 10 weeks of age.

Additionally, renal slides from the right kidneys of Sham animals were also subjected to PAS staining, photographed, and used to evaluate the glomerular size of each studied rat strain. Using the free software ImageJ (bundled with Java 8), we measured both the glomerular radius (r) and the glomerular area of 25 glomeruli from each studied animal, under a final 400× magnification. Using the radius information, we obtained the glomerular volume (V) in µm^3^, applying the following formula V = (4/3) × π × r^3^. Using the measurements obtained from the glomerular area analysis, and assuming the total number of nephrons in each kidney is equal, we calculated the total filtration area for each lineage, and then calculated the proportion by the adult animal’s weight in µm^2^/g.

### 2.5. Statistical Analysis

The results in the main text of this paper were presented as mean (M) ± standard error (SE) and (N), while in the Supplementary Material, results were presented as M ± standard deviation (SD) and (N). Data were analyzed by comparing groups, considering *p*-values under 0.05 as statistically significant. Prior to statistical analysis, all the parameters were verified through Shapiro–Wilk tests, to determine if the distribution of values were gaussian or not. We used Student’s *t*-test, Mann–Whitney, One-way or Two-way ANOVA, with Tukey’s multiple comparison post-test, according to the analyzed parameter. Calculations were performed using GraphPad Prism^®^ software, version 8.0.

## 3. Results

CKD was induced in rats through the surgical 5/6 renal ablation model, which is commonly associated with a mortality rate of around 20% of the Nx animals, in the first 30 days of follow-up. Corroborating the literature, our survival results presented in Table 1 show that Wistar rats submitted to the Nx model presented 17% of mortality during the study time. However, surprisingly, there were no deaths of either Lewis or Fischer Nx rats during the entire follow-up period.

**Figure 2:**
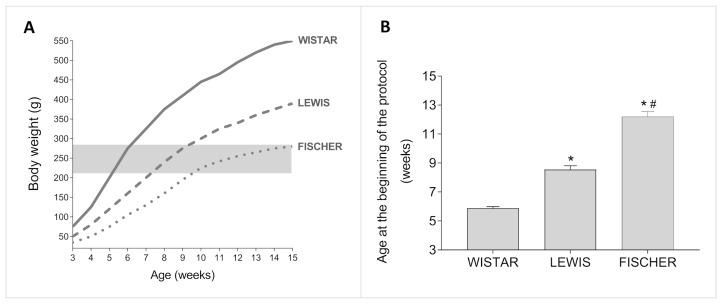
(**A**) Growth chart of the three studied rat strains. The highlighted rectangle represents the range of body weight in which the animals underwent 5/6 renal ablation for CKD induction. (**B**) Bar graph of the mean age, in weeks, in which the different strains of rats became eligible for 5/6 renal ablation, according to their body weight. One-way ANOVA, with Tukey’s multiple comparison test: *: *p* < 0.05 vs. Wistar, #: *p* < 0.05 vs. Lewis. (Non-Gaussian distribution, according to Shapiro–Wilk test).

In this study, 5/6 renal ablation was induced in rats weighing between 220 and 280 g, regardless of their age. Since the 3 rat strains employed in this study are known to present different growth rhythms, we used data from our Sham groups to build up an illustrative graph of the relation between body weight (BW) and age, in weeks, in each one of them. This data are presented in Figure 2A. As we can see, Wistar rats are eligible for 5/6 renal ablation surgery when they are between 5 and 6 weeks old. Lewis rats, in turn, achieve the required body weight for CKD induction when they are from 7 to 9 weeks old. Finally, Fischer rats can only be subjected to surgical 5/6 renal ablation when they are between 10 and 15 weeks old. As shown in Figure 2B, the Wistar rats subjected to the renal ablation model in this study were 5.9 ± 0.1 weeks old, while the Lewis animals were 8.5 ± 0.3 weeks old and the Fischer rats were 12.2 ± 0.3 weeks old, when submitted to the same procedure. It should be noted that such age differences among the rat strains in the beginning of the protocol were statistically significant.

**Figure 3:**
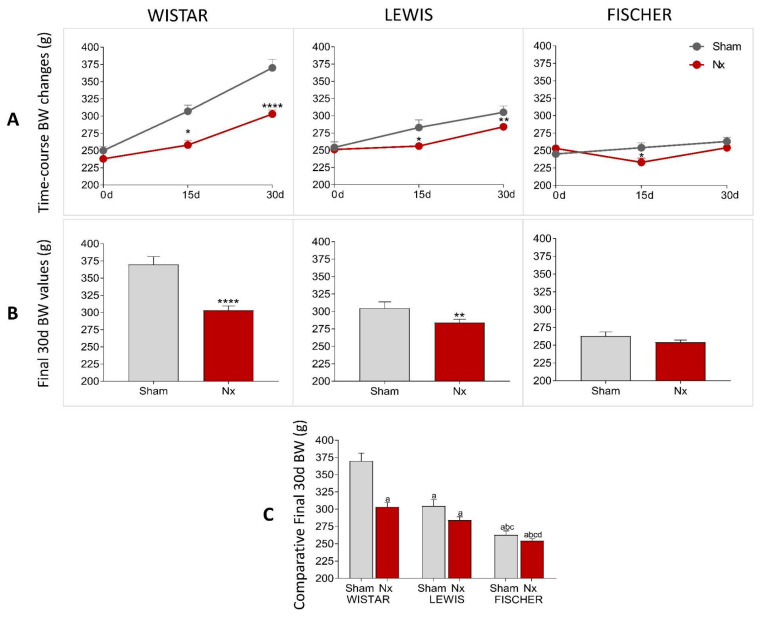
(**A**) Time-course BW changes (g) of all experimental groups, along the study period. (**B**) Body weight (g) of all experimental groups at end of the protocol. *: *p* < 0.05, **: *p* < 0.01, ***: *p* < 0.001, and ****: *p* < 0.0001 vs. respective Sham. (**C**) Comparison of final body weight (g) of the 3 different rat strains. One-way analysis of variance (ANOVA) was used to test for overall differences, followed by a post-hoc Tukey’s multiple comparison test to compare the means of all groups: a: *p* < 0.05 vs. Sham Wistar, b: *p* < 0.05 vs. Nx Wistar, c: *p* < 0.05 vs. Sham Lewis, d: *p* < 0.05 vs. Nx Lewis, e: *p* < 0.05 vs. Sham Fischer.

The BW of rats included in our protocol was monitored throughout the study period, as shown in Figure 3. On the first set of results, presented in Figure 3A, we showed the line graphs with the means of BW of animals from each experimental group, before CKD induction (preoperative moment), and 15 and 30 days after CKD induction. In Figure 3B, bar graphs of the final BW of each experimental group can be seen. As expected, Wistar, Lewis, and Fischer rats submitted to the 5/6 renal ablation model exhibited significantly lower weight gain compared to strain-matched Sham animals. Figure 3C illustrates the comparative analysis between Sham and Nx groups across the 3 rat strains. As observed, the final body weight of the control animals differed significantly among the strains, reflecting the intrinsic phenotypic characteristics of each lineage. Similarly, the final body weight of Lewis and Fischer Nx rats was significantly different from that observed in Wistar Nx animals, following a consistent pattern of inter-strain divergence.

**Figure 4:**
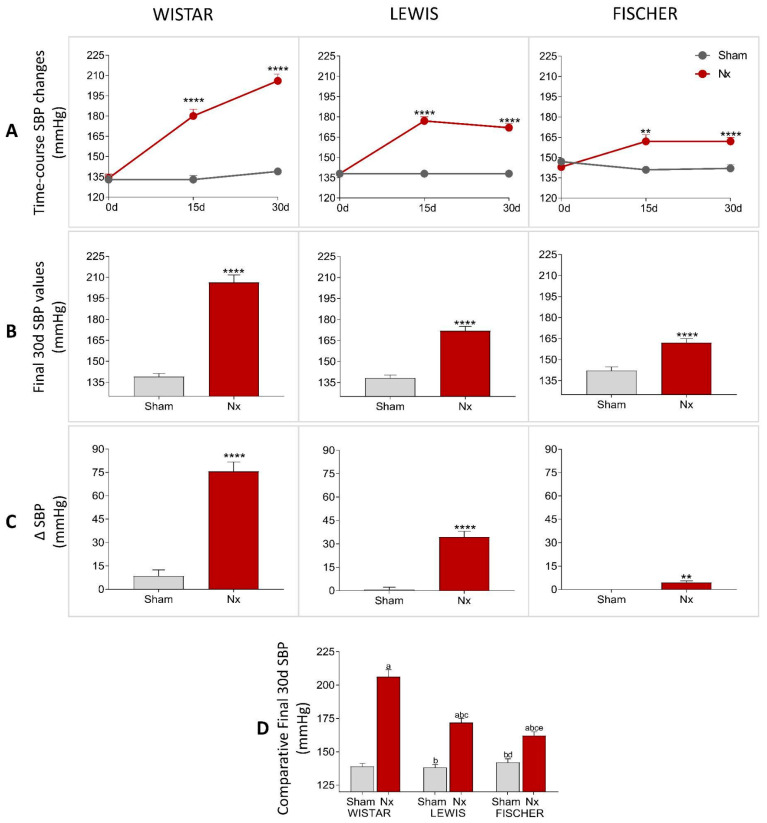
(**A**) Time-course SBP changes (mmHg) of all experimental groups during the study period. (**B**) SBP (mmHg) of all experimental groups, at the end of the protocol. (**C**) Delta graphs of SBP (mmHg), obtained by subtracting the values observed at 30 days from those observed at the beginning of the study. **: *p* < 0.01, ***: *p* < 0.001, and ****: *p* < 0.0001 vs. respective Sham. (**D**) Comparison of final SBP (mmHg) of the 3 different rat strains. One-way analysis of variance (ANOVA) was used to test for overall differences, followed by a post-hoc Tukey’s multiple comparison test to compare the means of all groups: a: *p* < 0.05 vs. Sham Wistar, b: *p* < 0.05 vs. Nx Wistar, c: *p* < 0.05 vs. Sham Lewis, d: *p* < 0.05 vs. Nx Lewis, e: *p* < 0.05 vs. Sham Fischer.

The systolic blood pressure of all animals included in the study was evaluated in the three main stages of the experimental protocol (0 d, 15 d, and 30 d). The results are illustrated as time-course line graphs in Figure 4A, bar graphs of the final results, in Figure 4B, and delta graphs of the means obtained by the subtraction of the initial SBP values from the final SBP values of each individual animal, as shown in Figure 4C. All rats subjected to 5/6 renal ablation rapidly evolved with significant hypertension after only 15 days of CKD induction. At this study point, Wistar Nx rats presented 181 ± 8 mmHg vs. 132 ± 3 mmHg observed in Wistar Sham rats, Lewis Nx exhibited 177 ± 3 mmHg vs. 138 ± 2 mmHg seen in Lewis Sham rats, and Fischer Nx presented 162 ± 5 mmHg vs. 141 ± 2 mmHg observed in Fischer Sham rats. Curiously, the rhythm of progression of hypertension associated with the Nx model, between 15 and 30 days of CKD induction, was extremely variable, according to the employed rat strain. While Wistar Nx rats presented a constant increase in SBP during the 30 days of the study, achieving a final value of 207 ± 5 mmHg, vs. 138 ± 2 mmHg in Wistar Sham, both Lewis and Fischer Nx animals maintained constant values of SBP between 15 and 30 days after CKD induction, achieving, respectively, 172 ± 3 mmHg in Lewis Nx, vs. 138 ± 2 mmHg in Lewis Sham, and 162 ± 3 mmHg in Fischer Nx, vs. 142 ± 3 mmHg in Fischer Sham. In Figure 4D, inter-strain comparison revealed that Sham groups from all the 3 strains exhibited comparable values, with no statistical differences observed. Conversely, when analyzing the Nx groups, while Wistar and Lewis rats showed similar levels of hypertension, the divergence between Wistar and Fischer rats reached statistical significance.

**Figure 5:**
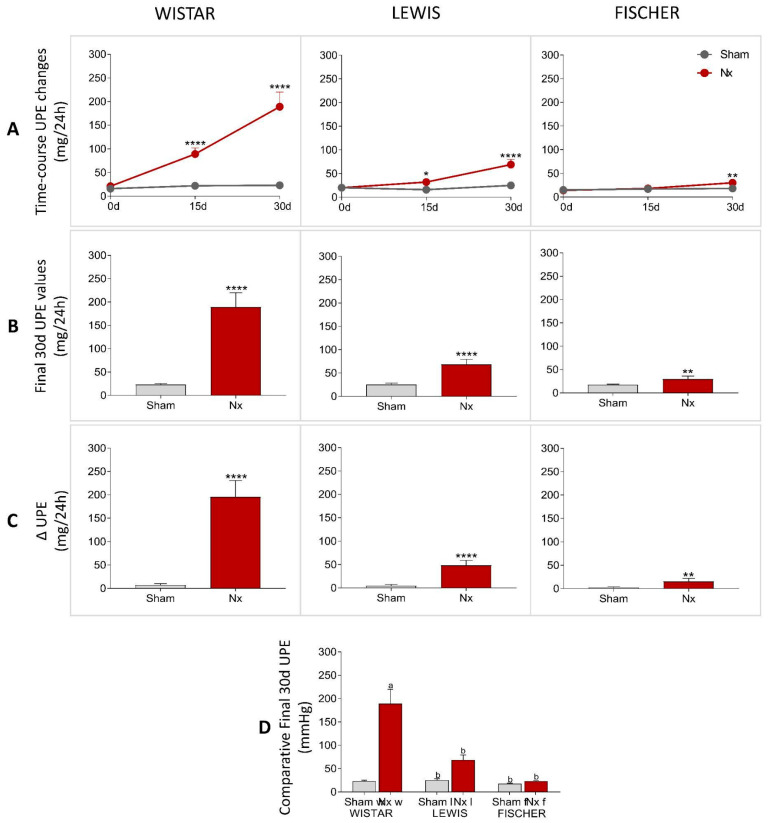
(**A**) Time-course UPE changes (mg/24 h) of all experimental groups during the study period. (**B**) UPE (mg/24 h) of all experimental groups at the end of the protocol. (**C**) Delta graphs of UPE (mg/24 h), obtained by subtracting the values observed at 30 days from those observed at the beginning of the study. *: *p* < 0.05, **: *p* < 0.01, ***: *p* < 0.001, and ****: *p* < 0.0001 vs. respective Sham. (**D**) Comparison of final UPE (mg/24 h) of the 3 different rat strains. One-way analysis of variance (ANOVA) was used to test for overall differences, followed by a post-hoc Tukey’s multiple comparison test to compare the means of all groups: a: *p* < 0.05 vs. Sham Wistar, b: *p* < 0.05 vs. Nx Wistar, c: *p* < 0.05 vs. Sham Lewis, d: *p* < 0.05 vs. Nx Lewis, e: *p* < 0.05 vs. Sham Fischer.

**Figure 6:**
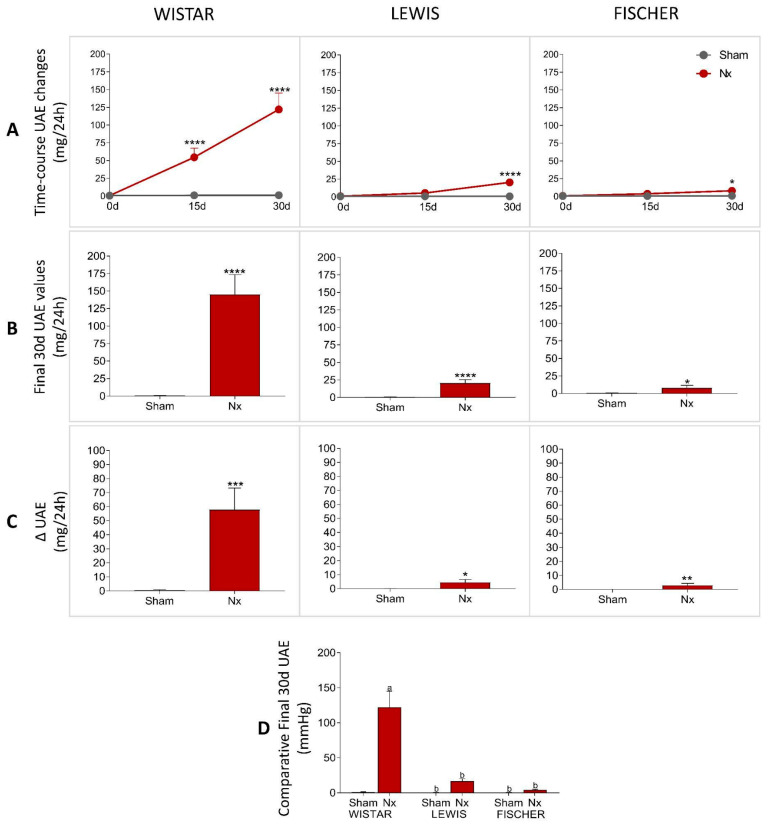
(**A**) Line graphs of the time-course UAE (mg/24 h) of all experimental groups during the study period. (**B**) UAE (mg/24 h) of all experimental groups at the end of the protocol. (**C**) Delta graphs of UAE (mg/24 h), obtained by subtracting the values observed at 30 days from those observed at the beginning of the study. *: *p* < 0.05, **: *p* < 0.01, ***: *p* < 0.001, and ****: *p* < 0.0001 vs. respective Sham. (**D**) Comparison of final UAE (mg/24 h) of the 3 different rat strains. One-way analysis of variance (ANOVA) was used to test for overall differences, followed by a post-hoc Tukey’s multiple comparison test to compare the means of all groups: a: *p* < 0.05 vs. Sham Wistar, b: *p* < 0.05 vs. Nx Wistar, c: *p* < 0.05 vs. Sham Lewis, d: *p* < 0.05 vs. Nx Lewis, e: *p* < 0.05 vs. Sham Fischer.

The urinary protein and albumin excretion of animals included in the study were assessed before the CKD induction (time 0), as well as 15 and 30 days after 5/6 renal ablation. UPE and UAE dosages performed over the course of the follow-up period were shown in line graphs on Figure 5A and Figure 6A, respectively, while the final values achieved by the end of the protocol are shown in bar graphs in Figure 5B and Figure 6B. Finally, the delta graphs of the same parameters are presented in Figure 5C and Figure 6C. Wistar Nx rats exhibited severe and significant proteinuria and albuminuria after only 15 days of CKD induction: 105 ± 17 and 73 ± 15 mg/24 h, vs. 24 ± 4 and 1 ± 1, in Wistar Sham. Between 15 and 30 days after renal ablation, both UPE and UAE frankly worsened in Nx Wistar rats, reaching, by the end of the study period, more than double the values observed at 15 days. After 30 days of Nx, Wistar rats exhibited 231 ± 40 mg/24 h of UPE and 145 ± 29 mg/24 h of UAE, compared to 23 ± 3 mg/24 h of UPE and 1 ± 1 mg/24 h of UAE, seen in Wistar Sham animals. Conversely, Lewis animals submitted to the Nx model presented only mild UPE and UAE after 15 days of CKD induction, compared to time-matched Lewis Sham: 32 ± 5 mg/24 h of UPE and 4 ± 1 mg/24 h of UAE, vs. 16 ± 3 mg/24 h of UPE and 1 ± 1 mg/24 h of UAE. Moreover, despite progressing with time, the UPE and UAE values exhibited by Lewis Nx after 30 days of CKD induction were much lower than those observed in time-matched Wistar Nx rats: At 30 days of follow-up, Lewis Nx showed 69 ± 11 mg/24 h of UPE and 17 ± 3 mg/24 h of UAE, compared to 25 ± 3 mg/24 h of UPE and 1 ± 1 mg/24 h of UAE in time-matched Lewis Sham. Finally, although submitted to the same surgical CKD induction, Fischer Nx animals did not develop significant levels of UPE or UAE during the study period: On the 15th day after 5/6 renal ablation, Fischer Nx animals exhibited 16 ± 1 mg/24 h of UPE and 2 ± 1 mg/24 h of UAE vs. 17 ± 1 mg/24 h of UPE and 1 ± 1 mg/24 h of UAE observed in Fischer Sham, while after 30 days of CKD induction, the Nx Fischer group shown 23 ± 1 mg/24 h of UPE and 4 ± 1 mg/24 h of UAE vs. 18 ± 1 mg/24 h of UPE and 1 ± 1 mg/24 h of UAE seen in Fischer Sham.

It is important to note that, despite being statistically different from the values observed in Sham animals of the same lineage, the UPE and UAE results of Fischer animals subjected to the renal ablation model can still be considered within the normal range for healthy animals. Inter-strain comparisons, as illustrated in Figure 5D and Figure 6D, show that for both parameters, no significant differences were observed among the Sham groups of the three strains, indicating comparable baseline values. However, within the Nx groups, Wistar rats exhibited significantly higher levels of both proteinuria and albuminuria compared to Lewis and Fischer rats. No statistical divergence was found between the two inbred strains. Complementary longitudinal analyses of these same parameters are shown in Supplementary Table S1.

**Figure 7:**
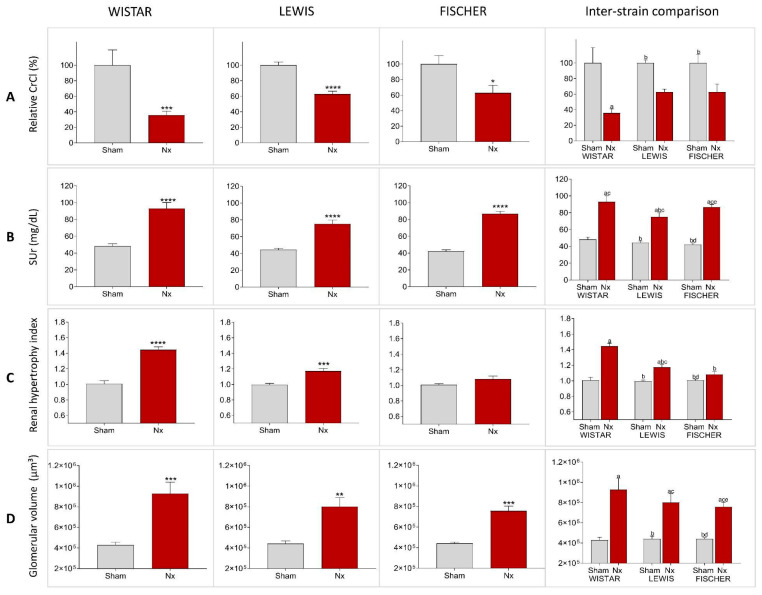
(**A**) Relative creatinine clearance (CrCl,%), (**B**) Serum urea (SUr, mg/dL), (**C**) Renal hypertrophy index and (**D**) Glomerular volume (μm^3^), after 30 days of CKD induction. Student’s T-test: *: *p* < 0.05, **: *p* < 0.01, ***: *p* < 0.001, and ****: *p* < 0.0001 vs. respective Sham. Inter-strain comparison: One-way analysis of variance (ANOVA) was used to test for overall differences, followed by a post-hoc Tukey’s multiple comparison test to compare the means of all groups: a: *p* < 0.05 vs. Sham Wistar, b: *p* < 0.05 vs. Nx Wistar, c: *p* < 0.05 vs. Sham Lewis, d: *p* < 0.05 vs. Nx Lewis, e: *p* < 0.05 vs. Sham Fischer.

Renal function analyses were performed after 30 days of CKD induction in all animals included in our study. The raw data of both the serum and urinary creatinine concentration, 24 h urinary volume, as well as the CrCl in mg/min/m^2^, can be seen in Table 1. In order to compare the reduction in CrCl after 30 days of CKD induction in the different rat strains, we performed a relative normalization, by considering the Sham CrCl of each animal strain as 100%. Consequently, the results of Nx rats are presented as the percentage change in relation to their respective Sham group. Bar graphs with these results are shown in Figure 7A. After 30 days of Nx, Wistar rats showed a severe decline in renal function, reaching only 42 ± 6% of the CrCl observed in strain-matched Sham animals, at the same time point. Conversely, both Lewis and Fischer animals underwent CKD induction exhibited a less prominent, but still significant, decrease in the CrCl rate, compared to their strain-matched Sham rats: 63 ± 4% and 63 ± 10%, respectively. Inter-strain comparisons revealed that the reduction in creatinine clearance (CrCl) was significantly more pronounced in Wistar Nx rats compared to both inbred strains. Despite the marked decline in the renal function observed in all rats submitted to 5/6 renal ablation, no statistical differences were found between Lewis Nx and Fischer Nx animals, which exhibited a comparable loss of renal function. On the other hand, serum urea analyses, as presented in Figure 7B, showed no differences among the 3 studied rat strains after 30 days of CKD induction; Wistar, Lewis, and Fischer Nx rats exhibited significant serum urea retention, compared to their respective Sham groups: 87 ± 10 vs. 51 ± 3, 75 ± 5 vs. 44 ± 2 and 87 ± 3 vs. 42 ± 2. Inter-strain comparisons demonstrated that all Sham groups exhibited similar levels of urea retention, with no significant differences observed. Interestingly, regarding the Nx groups, Lewis rats differed significantly only from the Wistar strain. No other significant differences were found in the remaining inter-strain comparisons.

To evaluate the adaptive hypertrophy of remnant renal mass in the different rat strains that underwent 5/6 renal ablation, left kidney weight (KW) of all animals was verified after 30 days of CKD induction, and the obtained values were divided by the BW of each respective animal. These quotients were multiplied by 10^3^, and the results for each experimental group are shown in Table 1. Since the studied rat strains present different basal kidney sizes, we calculated a kidney hypertrophy index by dividing the values obtained with the equation BW/KW*10^3^ of Nx animals by those obtained with the same equation of each respective Sham group. These results are presented in Figure 7C. Both Wistar and Lewis rats submitted to the 5/6 renal ablation model exhibited significant renal hypertrophy, compared to strain-matched Sham animals (*p* < 0.0001 and *p* < 0.001, respectively). Fischer Nx rats, in turn, did not develop compensatory renal hypertrophy compared to the Fischer Sham rats. Inter-strain comparisons show that both Lewis and Fischer Nx rats exhibited significantly lower hypertrophy indices when compared to Wistar Nx rats. It should be noted that no significant differences were observed between the two inbred strains.

Glomerular hypertrophy was evaluated in Nx animals through the analysis of the mean glomerular diameter (Mgd), measured in 25 glomeruli of each experimental animal. The obtained values of mGD of Wistar, Lewis, and Fischer Sham rats were very similar: 92 ± 3, 92 ± 2, and 93 ± 1, respectively (Table 2). After 30 days of 5/6 renal ablation, all rat strains presented increased values of mGD, as follows: 116 ± 5 in Wistar Nx, 112 ± 4 in Lewis Nx, and 110 ± 2 in Fischer Nx. Based on these values, we thus calculated the estimated glomerular volume in each rat strain. According to the bar graphs presented in Figure 7D, glomerular volume of Sham animals did not vary among the studied rat strains. However, regardless of the strain, all animals submitted to 5/6 renal ablation presented a significant increase in the glomerular volume, compared to their strain-matched Sham rats: 9.3 ± 1.1 × 10^5^ vs. 4.3 ± 0.3 × 10^5^ m3 in Wistar, 8.0 ± 0.9 × 10^5^ vs. 4.4 ± 0.3 × 10^5^ in Lewis, and 7.6 ± 0.5 × 10^5^ vs. 4.4 ± 0.1 × 10^5^ in Fischer rats. Inter-strain comparisons of glomerular hypertrophy revealed that Sham animals were very similar among the 3 studied rat lineages, and the same was observed for Nx rats.

**Figure 8:**
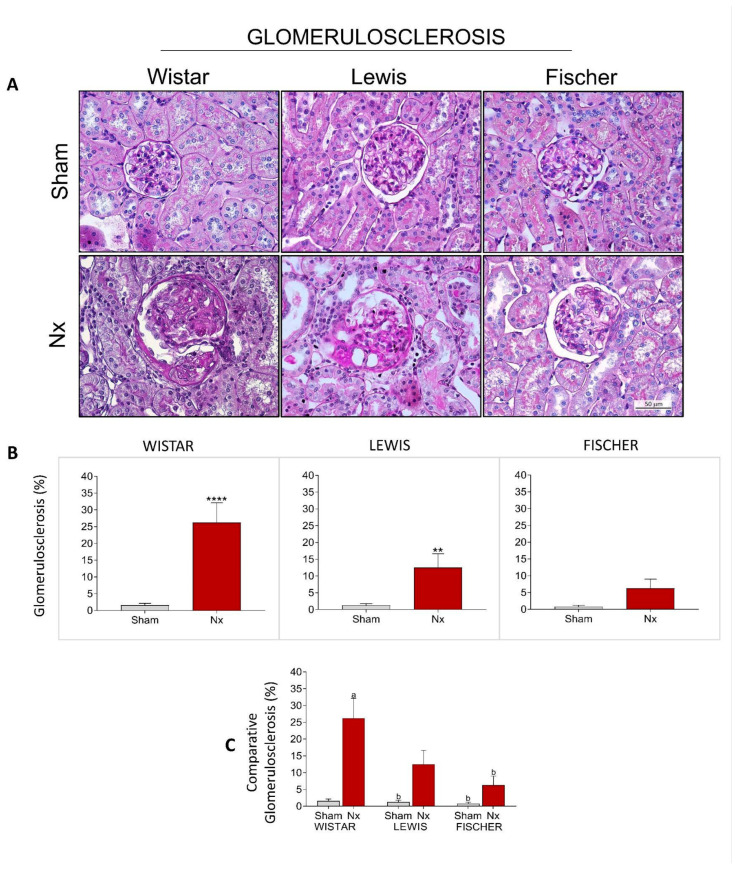
(**A**) Representative microphotograph panel of PAS-stained renal slides of each experimental group, under 400× magnification. (**B**) Bar graphs of glomerulosclerosis quantification (%). *: *p* < 0.05, **: *p* < 0.01, ***: *p* < 0.001, and ****: *p* < 0.0001 vs. respective Sham (**C**) Comparison of glomerulosclerosis (%) of the 3 different rat strains. One-way analysis of variance (ANOVA) was used to test for overall differences, followed by a post-hoc Tukey’s multiple comparison test to compare the means of all groups: a: *p* < 0.05 vs. Sham Wistar, b: *p* < 0.05 vs. Nx Wistar, c: *p* < 0.05 vs. Sham Lewis, d: *p* < 0.05 vs. Nx Lewis, e: *p* < 0.05 vs. Sham Fischer.

Structural glomerular damage was assessed in renal samples of all animals included in our study by staining the histological slides through the PAS method. Figure 8A shows a representative microphotograph panel of specimens of each experimental group. Based on the analysis of PAS slides, the percentage of glomerulosclerosis of each animal was calculated, and the results were presented as bar graphs in Figure 8B. As expected, no significant signs of glomerular damage were seen in Sham animals of any of the studied rat strains. Wistar Nx animals exhibited severe glomerulosclerosis after 30 days of CKD induction, with 27 ± 6% of affected glomeruli. Lewis Nx rats, in turn, presented less severe, but still significant glomerular lesion, exhibiting 13 ± 4% of affected glomeruli, after 30 days of renal ablation. Finally, Fischer Nx animals presented only 6 ± 1% of sclerotic glomeruli, after the same period of CKD follow-up. In Figure 8C, bar graphs of inter-strain comparisons show that, when subjected to Nx, both Lewis and Fischer animals developed less glomerulosclerosis than Wistar rats.

**Figure 9:**
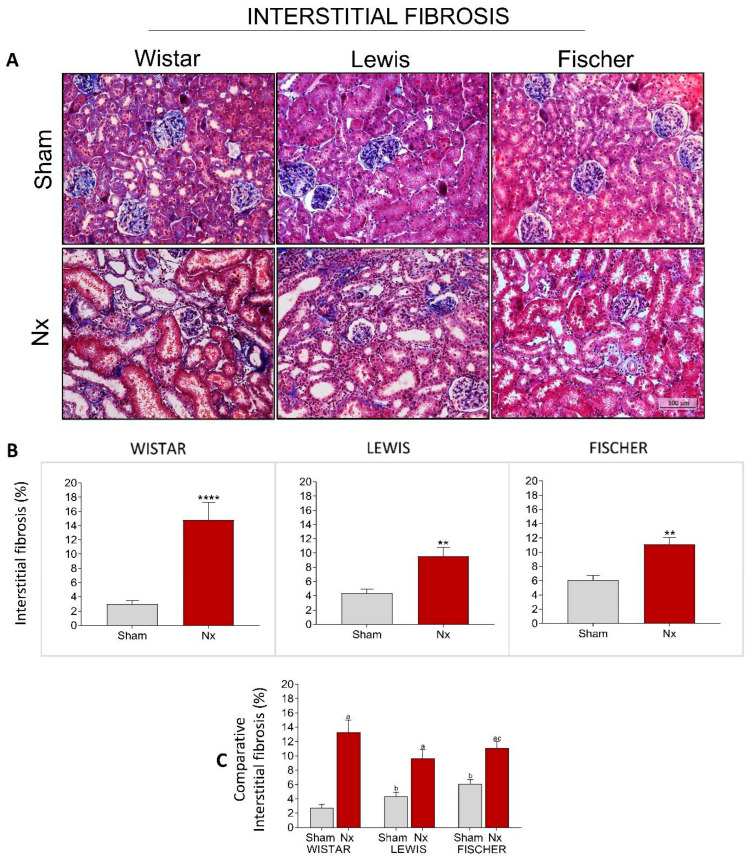
(**A**) Representative microphotograph panel of renal slides from each experimental group, submitted to the Masson trichrome staining technique, under 200× magnification. (**B**) Bar graphs of interstitial fibrosis quantification (%). *: *p* < 0.05, **: *p* < 0.01, ***: *p* < 0.001, and ****: *p* < 0.0001 vs. respective Sham. (**C**) Comparison of interstitial fibrosis (%) of the 3 different rat strains. One-way analysis of variance (ANOVA) was used to test for overall differences, followed by a post-hoc Tukey’s multiple comparison test to compare the means of all groups: a: *p* < 0.05 vs. Sham Wistar, b: *p* < 0.05 vs. Nx Wistar, c: *p* < 0.05 vs. Sham Lewis, d: *p* < 0.05 vs. Nx Lewis, e: *p* < 0.05 vs. Sham Fischer.

Interstitial expansion and fibrosis were evaluated in renal slides submitted to the Masson trichrome staining technique. Illustrative microphotographs of each experimental group can be seen in Figure 9A, while the quantitation of interstitial fibrosis, presented as the percentage of affected interstitial area, can be found in Figure 9B. Wistar Nx rats presented remarkable interstitial fibrosis after 30 days of CKD induction. Collagen deposition in this group was 5-fold greater than that observed in strain-matched Sham animals. On the other hand, both Lewis and Fischer Nx animals also exhibited significant renal fibrosis compared to their respective Sham groups; however, in these rat strains, interstitial collagen deposition was only around 2-fold greater than that observed in control animals. Further inter-strain comparisons are presented in Figure 9C. Interestingly, when compared to Wistar Sham rats, Fischer Sham animals exhibited significantly higher constitutive interstitial collagen content. However, for the Nx groups, no significant differences were observed among the three strains.

**Figure 10:**
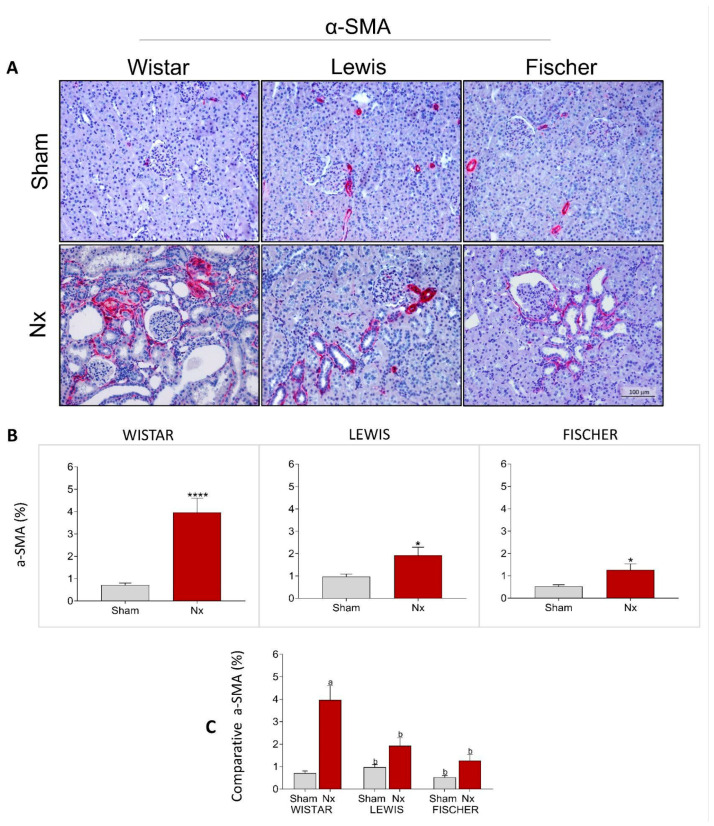
(**A**) Representative microphotograph panel of renal slides from each experimental group, submitted to imunohistochemistry for α-SMA, under 200× magnification, for myofibroblasts detection. (**B**) Bar graphs of the interstitial deposition of α-SMA (%). *: *p* < 0.05, **: *p* < 0.01, ***: *p* < 0.001, and ****: *p* < 0.0001 vs. respective Sham. (**C**) Comparison of α-SMA (%). of the 3 different rat strains. One-way analysis of variance (ANOVA) was used to test for overall differences, followed by a post-hoc Tukey’s multiple comparison test to compare the means of all groups: a: *p* < 0.05 vs. Sham Wistar, b: *p* < 0.05 vs. Nx Wistar, c: *p* < 0.05 vs. Sham Lewis, d: *p* < 0.05 vs. Nx Lewis, e: *p* < 0.05 vs. Sham Fischer.

Very similar results were observed when the presence of interstitial myofibroblasts was assessed by immunohistochemistry in the renal slides of animals from each experimental group. Myofibroblasts were identified through the deposition of interstitial smooth muscle α-actin (a-SMA), stained in red. Representative microphotographs of the experimental groups can be seen in Figure 10A, while the quantitation of the percentage of renal cortical area occupied by -SMA is presented as bar graphs in Figure 10B. After 30 days of CKD, Wistar animals presented 5-fold more interstitial α-SMA accumulation than that observed in strain-matched Sham rats. Conversely, both Lewis and Fischer Nx rats exhibited less prominent, but still significant, interstitial α-SMA accumulation. Nx rats of these strains presented only around 2-fold more myofibroblasts than those observed in the respective Sham groups. In Figure 10C, inter-strain comparisons show that Fischer Sham animals exhibited significantly lower basal interstitial α-SMA expression compared to Lewis rats. Regarding the Nx groups, both Lewis and Fischer rats showed significantly less α-SMA expression in the interstitial space when compared to Wistar animals.

**Figure 11:**
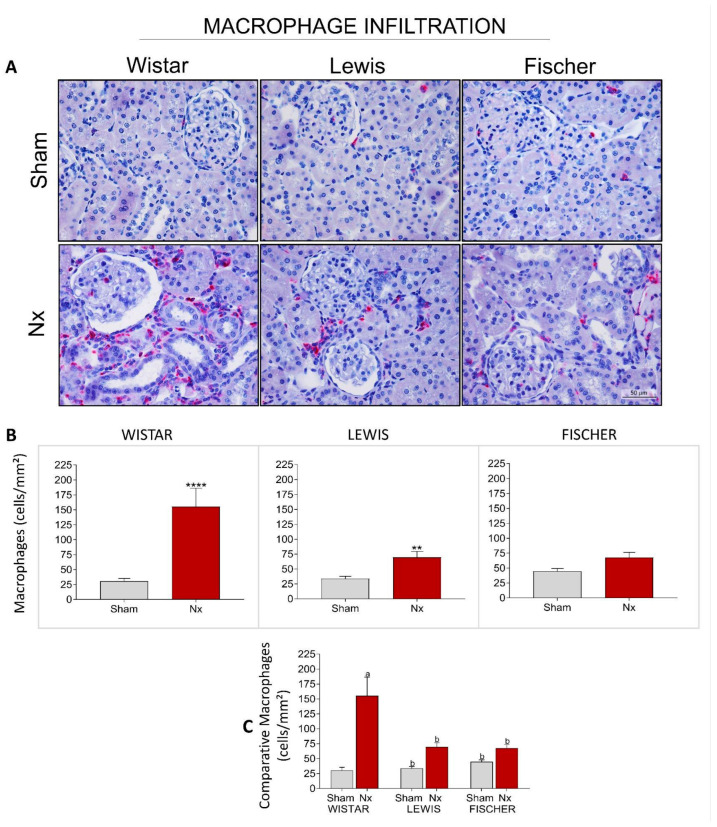
(**A**) Representative microphotograph panel of renal slides from each experimental group, submitted to imunohistochemistry for infiltrating macrophages (CD68+ cells), under 400× magnification. (**B**) Bar graphs of the quantification of tubulointerstitial infiltration by macrophages (CD68+ cells/mm^2^). *: *p* < 0.05, **: *p* < 0.01, ***: *p* < 0.001, and ****: *p* < 0.0001 vs. respective Sham. (**C**) Comparison of CD68+ cells (cells/mm^2^) of the 3 different rat strains. One-way analysis of variance (ANOVA) was used to test for overall differences, followed by a post-hoc Tukey’s multiple comparison test to compare the means of all groups: a: *p* < 0.05 vs. Sham Wistar, b: *p* < 0.05 vs. Nx Wistar, c: *p* < 0.05 vs. Sham Lewis, d: *p* < 0.05 vs. Nx Lewis, e: *p* < 0.05 vs. Sham Fischer.

Renal cortical interstitial inflammation was evaluated in the Nx rats through the presence of renal infiltrating macrophages (CD68+ cells), stained in red by immunohistochemistry. Illustrative microphotographs of renal slides from the different experimental groups can be seen in Figure 11A. The number of infiltrating CD68+ cells per mm^2^ were achieved, and the means of each group were presented in a bar graph in Figure 11B. Macrophage infiltration in Wistar Nx rats is significantly increased compared to strain-matched Sham: 178 ± 38 cells/mm^2^ vs. 25 ± 5 cells/mm^2^. This increase is considerably less expressive in Lewis rats submitted to 5/6 renal ablation, which presented means of 71 ± 10 cells/mm^2^ vs. 34 ± 4 cells/mm^2^ of the respective Sham group. Finally, Fischer Nx rats showed an even less severe increase: 68 ± 9 cells/mm^2^ vs. 45 ± 5 cells/mm^2^ in the Sham group. When comparing the Nx animals from the three different rat strains among each other (Figure 11C), it can be noted that both Lewis and Fischer Nx rats exhibited significantly lower interstitial macrophage infiltration compared to Wistar Nx animals.

**Figure 12:**
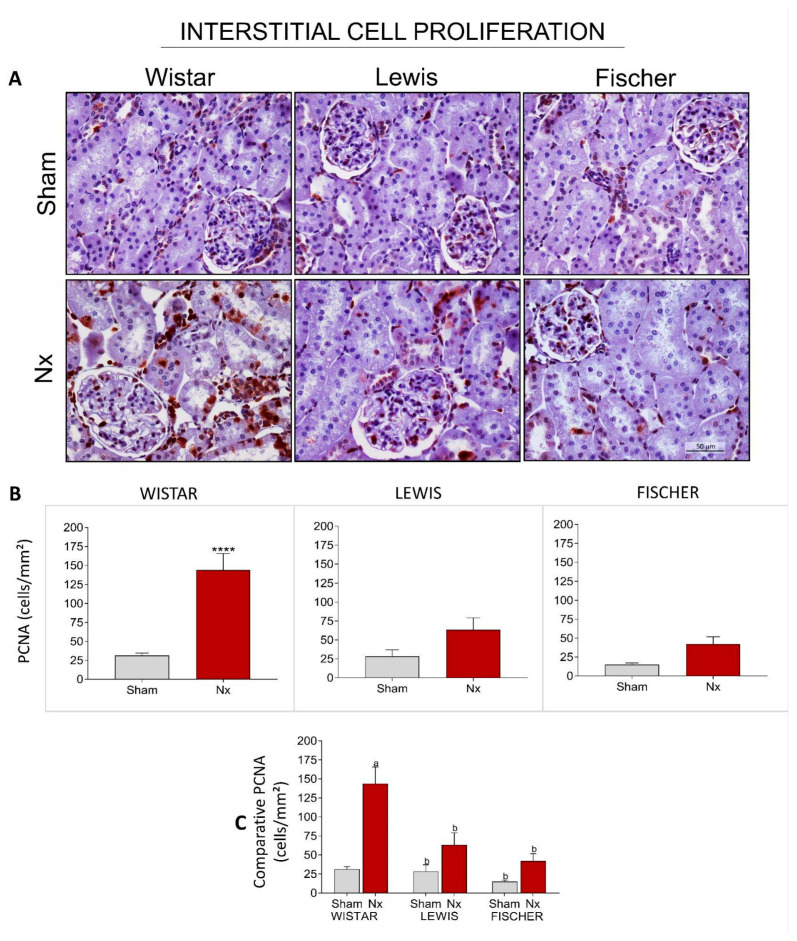
(**A**) Representative microphotograph panel of renal slides from each experimental group, submitted to imunohistochemestry for PCNA+ cells, under 400× magnification. (**B**) Bar graphs of the quantification of interstitial PCNA+ cells (cells/mm^2^). *: *p* < 0.05, **: *p* < 0.01, ***: *p* < 0.001, and ****: *p* < 0.0001 vs. respective Sham. (**C**) Comparison of PCNA+ cells (cells/mm^2^) of the 3 different rat strains. One-way analysis of variance (ANOVA) was used to test for overall differences, followed by a post-hoc Tukey’s multiple comparison test to compare the means of all groups: a: *p* < 0.05 vs. Sham Wistar, b: *p* < 0.05 vs. Nx Wistar, c: *p* < 0.05 vs. Sham Lewis, d: *p* < 0.05 vs. Nx Lewis, e: *p* < 0.05 vs. Sham Fischer.

For further evaluation of renal inflammation, we analyzed the presence of Proliferating Cell Nuclear Antigen (PCNA) in the interstitial space of the renal cortex in the different strains of rats. Figure 12A shows representative microphotographs of immunohistochemistry for PCNA in each group, and Figure 12B presents the graphs with the corresponding quantifications. Wistar Nx rats exhibited a significant increase in the interstitial cell proliferation rate, with 149 ± 27 cells/mm^2^ vs. 29 ± 4 cells/mm^2^ seen in the Wistar Sham group. Both Lewis and Fischer rats that underwent 5/6 renal ablation showed milder interstitial cell proliferation, compared to the Wistar strain. Lewis Nx animals presented 64 ± 16 interstitial PCNA+ cells/mm^2^ and Fischer Nx, in turn, exhibited only 42 ± 10 interstitial PCNA+ cells/mm^2^, compared to their respective Sham groups (28 ± 9 and 15 ± 2). Once more, when comparing the Nx animals from the three different rat strains among each other (Figure 12C), it can be noted that both Lewis and Fischer Nx rats exhibited a significantly lower number of renal cortical interstitial PCNA+ cells, compared to Wistar Nx rats.

The estimated total Nn exhibited by the Sham animals of each rat strain was achieved according to the steps illustrated in Figure 1, and the obtained raw values were presented in Table 2.

**Figure 13:**
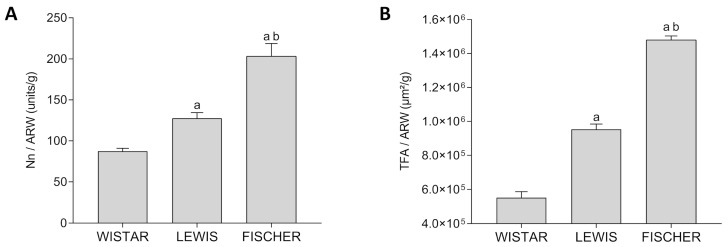
Relative Number of nephrons and glomerular filtration area in the different rat strains. (**A**) Nephron number corrected by the adult rat body weight (Nn/ARW, unids/g), in each studied rat strain (non-Gaussian distribution according to Shapiro–Wilk test). (**B**) Total glomerular filtration area corrected by the adult rat body weight (TFA/ARW, µm^2^/g) (Gaussian distribution). One-way ANOVA, with Tukey’s multiple comparison test: a: *p* < 0.05 vs. Wistar, b: *p* < 0.05 vs. Lewis.

In order to compare the significance of the obtained values of the total Nn among the different strains of rats, which exhibit different final body weights and, consequently, different absolute cardiac outputs to meet their different total body oxygen demands, we thus divided the Nn by the adult rat body weight (Nn/ARW, unids/g). These results were presented as bar graphs in Figure 13A. As shown, Wistar rats presented the lowest number of nephrons per gram of body weight among the 3 studied rat strains; only 87 ± 3 units/g. Lewis animals, in turn, presented a slightly higher number of nephrons per gram of body weight: 128 ± 7 units/g, which was significantly higher than that observed in Wistar rats. Finally, Fischer animals exhibited the highest number of nephrons per gram of body weight: 203 ± 15 units/g, which was statistically significant, when compared to both Wistar and Lewis strains.

Furthermore, in order to estimate the total glomerular filtration area in each studied rat strain (TFA), we performed the measurement of the glomerular area (mGA) of each experimental Sham group and thus multiplied this value by the obtained Nn of the respective rat strain. These results are shown in Table 2. Once more, we divided the obtained TFA by the adult rat body weight (TFA/ARW, m^2^/g), to compare the impact of the variances in the filtration area in face of the different size of the adult animals and the consequent total body demands of each rat strain. These quotients were presented as bar graphs in Figure 13B. As shown, Wistar rats presented the smallest TFA/ARW among the 3 studied strains: 6.2 × 10^5^ ± 2.6 × 10^4^ µm^2^/g. Lewis rats, in turn, exhibited a significantly bigger quotient of TFA/ARW (9.5 × 10^5^ ± 3.1 × 10^4^ µm^2^/g), when compared to the Wistar animals. Finally, Fischer rats presented the biggest quotient of TFA/ARW among the 3 studied rat lineages (1.5 × 10^6^ ± 2.3 × 10^4^ µm^2^/g), exhibiting statistical significance when compared to both Wistar and Lewis animals.

**Figure 14:**
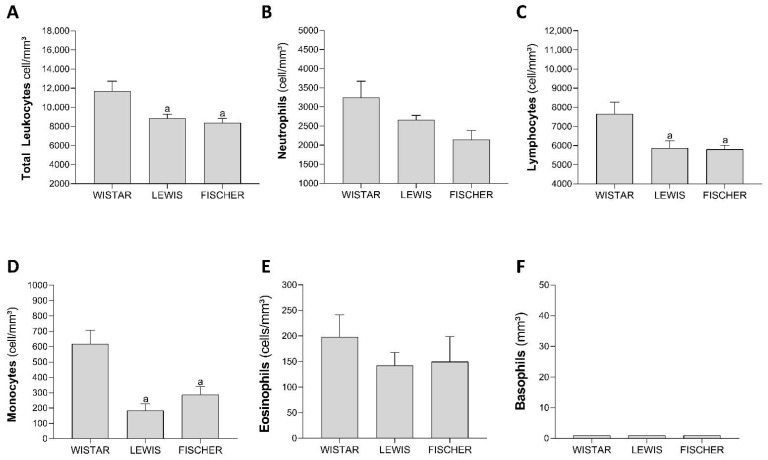
Leukogram of each rat strain (**A**) Total leukocyte (cell/mm^3^), (**B**) Neutrophils (cell/mm^3^), (**C**) Lymphocytes (cell/mm^3^), (**D**) Monocyte (cell/mm^3^), (**E**) Eosinophils (cell/mm^3^), and (**F**) Basophils (cell/mm^3^). One-way ANOVA, with Tukey’s multiple comparison test: a: *p* < 0.05 vs. Wistar, b: *p* < 0.05 vs. Lewis. (Gaussian distribution, according to Shapiro–Wilk test).

To further explore the mechanisms underlying the difference in CKD progression among the rat strains, a leukogram analysis was performed. As illustrated in the bar graphs of Figure 14A, the total leukocyte count for Wistar rats was 11,716 ± 1503, while Lewis and Fischer rats presented means of 8851 ± 624 and 8375 ± 714, respectively. Notably, both inbred strains exhibited significantly lower total leukocyte counts compared to the Wistar strain. Differential leukocyte counting was also performed and revealed that, compared to Wistar rats, both Lewis and Fischer animals presented numerically less neutrophils (2661 ± 176 and 2141 ± 368, vs. 3243 ± 637 in Wistar; Figure 14B), and statistically fewer peripheral lymphocytes (5864 ± 590 and 5797 ± 326, vs. 7658 ± 917 in Wistar; Figure 14C) and monocytes (185 ± 64 and 286 ± 85 vs. 617 ± 134 in Wistar; Figure 14D).

## 4. Discussion

The 5/6 nephrectomy in rats is a widely employed CKD model, used both to clarify unravel aspects of kidney pathophysiology and for the development and testing of new therapeutic strategies and drugs, designed to detain human CKD. According to the literature, the Nx model is one of the best ways to mimic human nephropathy features in rodents, since animals subjected to renal ablation commonly develop severe hypertension, increased cardiovascular risk, high mortality, pronounced proteinuria, and albuminuria, caused by the structural disruption of the glomerular filtration barrier, and renal histological changes, such as glomerulosclerosis and tubulointerstitial inflammation and fibrosis. Nonetheless, there are currently few references regarding the best rat strain to be employed for 5/6 renal ablation [13,15,32].

It is well-known that the gravity and the rate of CKD progression varies considerably according to the genetic background of patients, as well as to their lifestyle habits and even to environmental influences, among many other factors that are still unknown. Different patients affected by the same type of nephropathy, caused by the same etiology, may present completely distinct progression patterns of CKD. Thus, the characterization of CKD models, which evolve with different rhythms of renal function loss, may be of special interest to better resemble slow and rapidly progressive human nephropathy and to perform both quick and long-term drug tests [33,34].

In this study, we show that male albino rats from 3 different strains (Wistar, Lewis, and Fischer) of the same species (*Rattus norvegicus*), and with equivalent body weight, exhibited completely distinct biological responses and patterns of nephropathy progression when subjected to a highly standardized 5/6 renal ablation model. Corroborating the literature, Nx Wistar rats developed severe and rapidly progressive hypertension, associated with a mortality rate of 17%, mainly caused by cardiovascular events. Despite also presenting an initial increase in SBP, both Nx Lewis and Fischer animals exhibited no progression of this parameter between 15 and 30 days of follow-up, and no associated mortality. Interestingly, control Fischer rats are known to present higher baseline blood pressure, due to a hyper-reactive cardiovascular response to stress and a more anxious temperament, compared to both Wistar and Lewis animals, which make our findings with Fischer Nx even more significative [29,35,36,37,38,39].

Along with the development of malignant hypertension, corroborating previous studies, Wistar rats showed massive proteinuria and albuminuria, as soon as 15 days after renal ablation, which doubled after 30 days of Nx, reaching nephrotic levels by the end of the study period. Moreover, Wistar Nx animals exhibited exuberant compensatory remnant kidney hypertrophy and severe glomerulosclerosis after 30 days of CKD induction. Compensatory hyperfiltration in the remaining nephrons of an injured kidney usually occurs at the cost of developing glomerular hypertension. In conditions of renal mass loss (for example, after removal of a kidney or due to progressive kidney disease), the remaining nephrons attempt to compensate for the lost function. This process involves the vasodilation of afferent arterioles and the vasoconstriction of the efferent arterioles, resulting in an increase in intraglomerular blood pressure in the remaining nephrons [5]. This increase in pressure raises the individual glomerular filtration rate (GFR), a mechanism known as hyperfiltration, which helps to keep the total GFR of the kidney as close to normal as possible. Although this mechanism is beneficial in the short term for maintaining renal function, sustained glomerular hypertension and hyperfiltration are considered key factors that contribute to the progression of renal injury and sclerosis in the long term. Glomerular hypertension exerts mechanical stress on the glomerular endothelial walls, leading to endothelial lesion and to the establishment of local inflammation, with leukocyte migration, mesangial cell proliferation, extracellular matrix overproduction, and podocyte damage. Once injured, the glomerular filtration barrier loses its selectivity, leading to the glomerular excretion of macromolecules, such as albumin, to the tubular lumen. Abnormal protein concentration into the tubules can, itself, be a pro-inflammatory stimulus for the tubulointerstitial compartment, promoting the infiltration of inflammatory cells in the renal parenchyma, as well as increasing local fibroblasts proliferation and differentiation into myofibroblasts, which, in turn, lead to exacerbated extracellular matrix deposition and to the development of tubulointerstitial fibrosis. Accordingly, Wistar rats subjected to the Nx model exhibited significant tubulointerstitial macrophage infiltration, increased interstitial cell proliferation, myofibroblasts accumulation, and significant interstitial fibrosis deposition [4,5,40,41,42].

Surprisingly, Lewis Nx rats presented only mild proteinuria and albuminuria, and Fischer Nx animals did not even develop any alteration in the UPE and UAE levels, compared to the controls. Similar findings were observed regarding renal hypertrophy and glomerulosclerosis. Consequently, even under the same injury inflicted on Wistar rats by the 5/6 renal mass reduction, Lewis, and in particular, Fischer rats developed less or no renal interstitial inflammation and fibrosis.

Such discrepancy in the biological response of the different studied rat strains to the Nx model observed in our study can be related to the difference in the age of the animals when subjected to CKD induction. Since Wistar, Lewis, and Fisher rat lineages exhibit different growth curves, rats from these strains were submitted to 5/6 renal ablation at different ages. Wistar rats became eligible for the experimental model at about 42 days (5 weeks) of life, when, although sexually mature, these animals can still be considered as preadolescents at the beginning of their growth spurt. On the other hand, Lewis rats achieve the required body weight for Nx surgery after 63 days (9 weeks) of birth, and Fischer animals only reach the same body weight when they are 105 days (12 weeks) old. This means that both isogenic lineages underwent renal ablation only after the complete body development, when they can already be considered young adults. According to comparisons between classical studies employing the 5/6 renal ablation model, the age at which CKD is induced in the animals may change the development rhythm of the nephropathy. Tain and collaborators suggested that very young Sprague–Dawley animals (1–2 weeks of age) submitted to renal ablation rapidly developed more severe hypertension, creatinine, and urea retention, compared to adult animals [43,44,45,46].

The age difference among the studied rat strains by the time of CKD induction represents the biggest limitation of our study. Once the three rat lineages exhibit different growth curves, we were forced to choose between inducing CKD in animals with the same body weight or with the same age, and it was not an easy decision to take, since we understand that both choices could influence the obtained results and invite further criticism. However, our choice of choosing body weight instead of age as the main baseline parameter in this study was based in the following aspects: First of all, despite being almost 6 weeks younger than Fischer rats, at the beginning of the study period, Wistar animals were already considered sexually mature, which means that, in general lines, all three different rat strains can be considered adults when subjected to 5/6 renal ablation. It is true that, unlike for human beings, rat nephrogenesis is a continuous process that extends from the fetal period until approximately 7 to 10 days after birth (postnatal), the period in which the final number of glomeruli that will rest until adulthood is defined. However, it is also true that the whole progress of nephrogenesis is already complete at 6 weeks of age, which means that the differences in the basal nephron number among the three rat strains observed in this study are not likely to be related to the age differences at the beginning of the protocol [47]. Another important point that was considered when choosing an unnegotiable range of basal body weight (220 and 280 g) to induce CKD by the 5/6 renal ablation regards the accuracy and safety of the surgery. Standardization by body weight was prioritized to ensure the homogeneity of the surgical technique, since body size correlates more precisely with renal volume and vessel caliber than age, being a critical parameter for the accuracy of renal ablation and the rigorous adjustment of anesthetic and analgesic doses. Moreover, regardless of the employed rat strain, most articles in the literature that use surgically induced CKD models start with animals weighing between 200 and 300 g, because of the influence exerted by the body weight at the time of surgery as a crucial feature to keep lower mortality rates: Lower body weight (under 150 g, for instance) is a risk factor for poor surgical outcomes, frequently associated with infections, dehydration, and deficits in wound healing related to insufficient energy reserve to support the metabolic stress of the post-surgical recovery period. On the other hand, higher body weight (over 300 g) in laboratory rats is generally related to a higher amount of adipose tissue, which directly impairs access and manipulation of intra-abdominal organs and increases intra-operatory bleeding [23,25,48].

Beyond the age differences, another very reasonable explanation for the renoprotection exhibited by Lewis and, particularly, by Fischer rats, compared to Wistar animals could be a higher basal number of nephrons. It is widely known that the number of nephrons at birth is strongly related to a higher or lower predisposition to develop CKD in adulthood. People with kidneys considered small relative to their body mass have a higher incidence of hypertension and albuminuria, suggesting that these individuals have a smaller number of nephrons and, consequently, deal with renal overload, even in the absence of an initial renal injury [49,50]. Here, we demonstrated that Lewis and Fischer rats have, respectively, 1.4× and 2.3× more nephrons per gram of body weight in adulthood, compared to Wistar rats, which may have significantly contributed to the resistance of these rats in developing and progressing CKD. It is possible that the reduction by 5/6 in renal mass of Fischer rats had a smaller effect on the overall renal function of these animals, due to their greater basal number of nephrons and total filtration area.

Moreover, another potential factor that may have contributed to the different inflammatory response observed across the three studied rat strains is their distinct baseline hematological profiles. Corroborating data provided by Charles River Laboratories, our leukogram analysis showed that there are considerable differences in circulating white blood cells amount Wistar, Lewis, and Fischer rats. Of note, Wistar animals exhibit higher baseline number of circulating neutrophils, lymphocytes, and monocytes compared to both Lewis and Fischer strains. Moreover, Fischer animals present the lowest total leukocyte counts among the three lineages, which may partially explain the milder and more limited immune response in these rats and, consequently, the development of less expressive tissue inflammation. Inherent differences in the immune cell repertoire likely influence the magnitude of the systemic inflammatory response in experimental models. In the specific case of Fischer rats, this milder immune response may be further reinforced by their hyper-responsive hypothalamic-pituitary-adrenal (HPA) axis; the resulting higher basal corticosterone levels are known to exert an immunosuppressive effect, limiting leukocyte proliferation and recruitment. Although the singular hematological profile for each strain is a promising explanation for their response, we cannot discard the possibility that the higher nephron numbers are a crucial factor for the presented results [51,52,53,54,55].

## 5. Conclusions

Based on the above, we can conclude that, despite being submitted to the same surgical procedure, Wistar, Lewis, and Fischer rats exhibit different patterns of CKD progression and achieve distinct degrees of renal function loss, after undergoing 5/6 renal ablation. If applied to Wistar rats, Nx is an excellent model for mimicking rapidly progressive, severe CKD and for quick drug and therapy tests, designed for advanced stages of human nephropathy. When Lewis rats are chosen as target animals for 5/6 renal ablation, slower CKD progression and less severe resulting nephropathy can be expected, which can be especially useful to simulate intermediate degrees of human CKD and allow multiple interventional tests during longer study periods. Finally, it is important to highlight that, when using Fischer rats, the 5/6 renal ablation model described in this paper can be insufficient to promote significant renal damage or to resemble human nephropathy within a 30-day follow-up period. Additional interventions, such as the pharmacological chronic nitric oxide synthesis inhibition or the adoption of a high-salt diet, as well as longer periods of follow-up may be required in order to promote human-like CKD in this rat strain. The significant renoprotection observed in Fischer rats probably derives from a set of strain-specific features, which include a bigger number of nephrons at birth, associated with a larger glomerular area, smaller body weight in adulthood, and less susceptible to inflammation, due to the reduced basal number of peripheral and tissue leukocytes.

## Figures and Tables

**Table 1:** Survival rate during the experimental period (%); kidney weight (KW; g); kidney weight ratio: kidney weight divided by the individual body weight and multiplied by 10^3^ (KW/BW*10^3^), Serum creatinine concentration (SCr; mg/dL); urinary creatinine concentration (Ucr; mg/dL); urinary volume (UV; mL/24 h); and creatinine clearance (CrCl; mg/min/m2). ^a^: *p* < 0.05 vs. Sham Wistar, ^b^: *p* < 0.05 vs. Nx Wistar, ^c^: *p* < 0.05 vs. Sham Lewis, ^d^: *p* < 0.05 vs. Nx Lewis, ^e^: *p* < 0.05 vs. Sham Fischer.

	Sham Wistar	Nx Wistar	Sham Lewis	Nx Lewis	Sham Fischer	Nx Fischer
Survival rate (%)	100%	83%	100%	100%	100%	100%
KW (g)	1.4 ± 0.1	1.7 ± 0.0 ^a^	0.9 ± 0.0 ^ab^	1.1 ± 0.0 ^ab^	1.0 ± 0.0 ^ab^	1.0 ± 0.0 ^ab^
KW/BW × 10^3^	3.7 ± 0.2	5.4 ± 0.1 ^a^	3.2 ± 0.1 ^ab^	3.8 ± 0.1 ^bc^	3.5 ± 0.1 ^b^	3.8 ± 0.1 ^bc^
SCr (mg/dL)	0.59 ± 0.05	0.99 ± 0.13 ^a^	0.48 ± 0.01 ^b^	0.76 ± 0.03 ^abc^	0.46 ± 0.03 ^bd^	0.75 ± 0.05 ^abce^
UCr (mg/dL)	69 ± 8	21 ± 3 ^a^	132 ± 9 ^ab^	48 ± 4 ^c^	128 ± 11 ^abd^	39 ± 6 ^ce^
UV 30d (mL/24 h)	19 ± 8	47 ± 4 ^a^	10 ± 1 ^ab^	26 ± 2 ^bc^	8 ± 1 ^abd^	26 ± 2 ^bce^
CrCl (mg/min/m^2^)	57 ± 11	18 ± 4 ^a^	41 ± 4 ^b^	28 ± 4	36 ± 4	22 ± 4

**Table 2:** Total Nephron number (Nn; units) achieved according to the steps illustrated in Figure 1. Adult rat body weight (ARW, g): mean body weight of each rat strain at 10 weeks of age. Mean glomerular diameter, (mGD, µm), mean glomerular area (mGA, µm^2^), and total filtration area (TFA; µm^2^). ^a^: *p* < 0.05 vs. Wistar, ^b^: *p* < 0.05 vs. Lewis.

	Wistar Sham	Lewis Sham	Fischer Sham
Nn (units)	3.9 × 10^4^ ± 1.5 × 10^3^	3.5 × 10^4^ ± 1.8 × 10^3^	3.9 × 10^4^ ± 2.9 × 10^3^
ARW (g)	450	272 ^a^	190 ^a,b^
mGD (µm)	92 ± 3	92 ± 2	93 ± 1
mGA (µm^2^)	7.1 × 10^3^ ± 3.0 × 10^2^	7.5 × 10^3^ ± 2.4 × 10^2^ a	7.3 × 10^3^ ± 1.1 × 10^2 a^
TFA (µm^2^)	2.8 × 10^8^ ± 1.2 × 10^7^	2.6 × 10^8^ ± 8.6 × 10^6^	2.8 × 10^8^ ± 4.4 × 10^6^

## Data Availability

The raw data supporting the conclusions of this article will be made available by the authors on request.

## Supplementary Materials

The following supporting information can be downloaded at: https://www.mdpi.com/article/10.3390/life16030420/s1, Table S1: Time course results.
