# Supplementary material for: Differences on the Natural Course of Chronic Kidney Disease Progression, Induced by 5/6 Renal Ablation Model in Three Different Rat Stains: Wistar, Lewis, and Fischer 344
Source: Life (Basel). 2026 Mar 4;16(3):420. doi: 10.3390/life16030420 (PMC13028075; doi:10.3390/life16030420)
Supplement: Supplementary file 1 [file life-16-00420-s001.zip › life-4090735-supplementary.pdf]

Supplementary Table S1: Time-course Mean±1SD (N) of BW (g), SBP (mmHg), UPE (mg/24h) and UAE (mg/24h) values in all experimental groups, along the study period. Two-way ANOVA with factors "group (Sham vs Nx)" and "time (0, 15, 30 days)", followed by Tukey's post hoc test. p<0.05vs: a: Sham 0d, b: Sham 15d, c: Sham 30d, d: Nx 0d, and e: Nx 15d, of each respective rat strain.

		BW	SBP	UPE	UAE
WISTAR	Sham 0d	250 ± SD (N)	133 ± 3	16 ± 2	0.50 ± 0.15
	Sham 15d	307 ± 9[a]	133 ± 3	22 ± 2	0.96 ± 0.23
	Sham 30d	370 ± 12[ab]	139 ± 3	23 ± 2	1.13 ± 0.30
	Nx 0d	238 ± 3[bc]	134 ± 3	21 ± 3	0.59 ± 0.33
	Nx 15d	258 ± 7[bc]	180 ± 5[abcd]	89 ± 13[abcd]	54.55 ± 13.06[abcd]
	Nx 30d	303 ± 6[acbe]	206 ± 5[abcde]	189 ± 31[abcde]	122.03 ± 23.08[abcde]
LEWIS	Sham 0d	257 ± 7	138 ± 2	20 ± 3	0.70 ± 0.21
	Sham 15d	283 ± 11	138 ± 2	16 ± 3	0.77 ± 0.20
	Sham 30d	305 ± 9[a]	138 ± 2	25 ± 3	0.68 ± 0.22
	Nx 0d	251 ± 5[bc]	136 ± 1	20 ± 1	0.96 ± 0.16
	Nx 15d	256 ± 5[c]	177 ± 3[abcd]	32 ± 5	3.67 ± 1.15
	Nx 30d	284 ± 5[ade]	172 ± 3[abcd]	69 ± 11[abcde]	16.58 ± 3.35[abcde]
FISCHER	Sham 0d	245 ± 5	147 ± 2	15 ± 1	0.50 ± 0.08
	Sham 15d	254 ± 7	141 ± 2	17 ± 1	0.50 ± 0.08
	Sham 30d	263 ± 6	143 ± 3	18 ± 1	0.70 ± 0.14
	Nx 0d	253 ± 5	143 ± 2	14 ± 1	0.69 ± 0.10
	Nx 15d	233 ± 6[c]	162 ± 5[abcd]	16 ± 1	2.21 ± 0.43
	Nx 30d	254 ± 3	162 ± 3[abcd]	23 ± 1	4.02 ± 1.16
